# TRAINING OLDER ADULTS IN THE USE OF INTERNET TECHNOLOGY

**DOI:** 10.1093/geroni/igae098.2569

**Published:** 2024-12-31

**Authors:** Donna Case, Sheryl Groden, Michelle Sahli

**Affiliations:** University of Michigan Flint, Flint, Michigan, United States; University of MIchigan Flint, Flint, Michigan, United States; University of MIchigan Flint, Flint, Michigan, United States

## Abstract

This study was conducted to investigate whether small group (2:1) technology training increases the frequency of technology use among older adults and how these older adults perceived this training. Graduate occupational therapy students, under faculty supervision, provided 4 weekly technology tutoring sessions to older adults at a senior center and an independent living facility located in Genesee County, MI. Participants received training in pairs. This training consisted of internet safety and additional instruction centered on participants’ interests. When requested, participants were offered assistive devices to help them use technology. Pre and post surveys were administered to participants to examine their independent technology use and attitudes towards the training sessions. Students provided training to 31 older adults with only 3 participants requesting assistive devices. The surveys showed an increase in the proportion of participants independently using technology for 10 out of 11 activities; however, these results were not statistically significant. Analysis of qualitative data revealed that most participants felt comfortable using technology post intervention and that they learned more about technology. Participants also liked the frequency and length of the sessions and reported forming relationships with others during the sessions. Although not statistically significant, perhaps due to small sample size, these findings provide evidence that training in small groups provided by students may increase technology use by older adults and that this type of training is acceptable to older adults. Future studies should be conducted in larger samples and with other populations to further explore this type of training program.

